# Chemical Composition and Antioxidant Properties of Juniper Berry (*Juniperus communis* L.) Essential Oil. Action of the Essential Oil on the Antioxidant Protection of *Saccharomyces cerevisiae* Model Organism

**DOI:** 10.3390/antiox3010081

**Published:** 2014-02-24

**Authors:** Martina Höferl, Ivanka Stoilova, Erich Schmidt, Jürgen Wanner, Leopold Jirovetz, Dora Trifonova, Lutsian Krastev, Albert Krastanov

**Affiliations:** 1 Department of Pharmaceutical Chemistry, Division of Clinical Pharmacy and Diagnostics, University of Vienna, Vienna 1090, Austria; E-Mails: info@artandfragrance.de (E.S.); leopold.jirovetz@univie.ac.at (L.J.); 2 Department Biotechnology, University of Food Technologies, Plovdiv 4002, Bulgaria; E-Mails: wstoilowa@yahoo.com (I.S.); dora.trifonova@gmail.com (D.T.); a_krastanov@uft-plovdiv.bg (A.K.); 3 Kurt Kitzing Co., Wallerstein 86757, Germany; E-Mail: juergen.wanner@kurtkitzing.de; 4 University Laboratory for Food Analyses, University of Food Technologies, Plovdiv 4002, Bulgaria; E-Mail: loutcian@abv.bg

**Keywords:** juniper essential oil, *Juniperus communis*, GC/MS, antioxidant, *Saccharomyces cerevisiae*, antioxidant enzymes

## Abstract

The essential oil of juniper berries (*Juniperus communis* L., Cupressaceae) is traditionally used for medicinal and flavoring purposes. As elucidated by gas chromatography/flame ionization detector (GC/FID) and gas chromatography/mass spectrometry (GC/MS methods), the juniper berry oil from Bulgaria is largely comprised of monoterpene hydrocarbons such as α-pinene (51.4%), myrcene (8.3%), sabinene (5.8%), limonene (5.1%) and β-pinene (5.0%). The antioxidant capacity of the essential oil was evaluated *in vitro* by 2,2-Diphenyl-1-picrylhydrazyl (DPPH) scavenging, 2,2-azino-bis-3-ethylbenzothiazoline-6 sulfonic acid (ABTS) radical cation scavenging, hydroxyl radical (ОН^•^) scavenging and chelating capacity, superoxide radical (^•^O_2_^−^) scavenging and xanthine oxidase inhibitory effects, hydrogen peroxide scavenging. The antioxidant activity of the oil attributable to electron transfer made juniper berry essential oil a strong antioxidant, whereas the antioxidant activity attributable to hydrogen atom transfer was lower. Lipid peroxidation inhibition by the essential oil in both stages, *i.e.*, hydroperoxide formation and malondialdehyde formation, was less efficient than the inhibition by butylated hydroxytoluene (BHT). *In vivo* studies confirmed these effects of the oil which created the possibility of blocking the oxidation processes in yeast cells by increasing activity of the antioxidant enzymes superoxide dismutase (SOD), catalase (CAT), and glutathione peroxidase (GPx).

## 1. Introduction

Reactive oxygen species (ROS) such as Н_2_О_2_, ^•^O_2_^–^ and ОН^•^ are produced in the organisms during cellular metabolism. At lower concentrations they participate in cellular physiological reactions [1]. Their overproduction, however, largely determines cell survival. The ROS inactivation and removal depends on non-enzymatic and enzymatic protective mechanisms. Research on ROS-induced damage has shown that antioxidant production is genetically controlled in the cells [2]. The focus on antioxidants naturally contained in essential oils is directly related to their application aimed at the prevention of oxidative damage to biological systems by ROS. Low-molecular antioxidants can enhance organism stability under oxidative stress [3].

For centuries, juniper berries have been used in folk medicine for the treatment of opportunistic infections, as a spice for meat, and as flavor in the preparation of gin and raki [4,5].

The antioxidant activity of essential oils from different juniper berry species has been established *in vitro* [6]. Anti-radical activity depends on the oil components, *i.e.*, their chemical nature and concentration [7,8,9,10]. Regardless of the differences in the composition of juniper berry essential oils, they are dominated by terpene hydrocarbons.

In many cases, the essential oil antioxidant activity cannot be attributed to the dominant compounds α- and β-pinene. These monoterpene hydrocarbons in juniper berry essential oil do not contribute to a significant inhibition of malondialdehyde formation [9]. The carriers of antioxidant properties in relation to lipid peroxidation in both its stages are α- and γ-terpinenes and, to a significantly lesser extent, their sesquiterpene analogues. This has been established both for juniper essential oils [7,8,9] and for pure terpene hydrocarbons: terpinolene, α-terpinene and γ-terpinene [10]. Myrcene, α- and β-pinene only inhibit lipid peroxidation in the second stage; sabinene, limonene, α-pinene, and myrcene demonstrate anti-radical activity in relation to DPPH radical [11,12]. The scavenging effect of ОН^•^ and the protection of deoxyribose against degradation are mainly due to β-pinene and limonene [6]; the ^•^O_2_^–^ neutralization is determined by germacrene-D [13]. The 10-membered ring system and the three double bonds acting as electron-rich centers in germacrene-D determine its anti-radical activity.

A number of studies have shown that the monoterpene components also contained in juniper essential oil enhance, through their antioxidant activity, the oxidative stress resistance of living organisms. Their antiradical activity affects the levels of the most important enzymes responsible for the neutralization of ROS: SOD, CATs, peroxidases, and glutathione transferase [12,14,15]. *Saccharomyces cerevisiae* is widely used for the better understanding of the cellular protection against ROS. Its enzymatic anti-ROS antioxidant protection has been well studied [16,17,18,19,20]. In this aspect, it was interesting to study the possibility of increasing the antioxidant protection of yeast cells using juniper berry essential oil and oxidant detoxification *in vivo*. The antioxidant properties of the essential oil both *in vitro* and *in vivo* are important for the overall evaluation of its action.

The aims of the present study were to investigate the chemical composition of the essential oil of juniper berries, to assess *in vitro* the antioxidant activity of juniper berry essential oil and to prove *in vivo* its preventive effect upon the oxidative damage in *S. cerevisiae* due to its action on the antioxidant enzymes SOD, CAT and GPx.

## 2. Experimental Section

### 2.1. Materials

Essential oil of juniper berries (*Juniperus communis* L., Cupressaceae) is a commercial product from Bulgaria. DPPH, β-Nicotinamide adenine dinucleotide 2-phosphate reduced tetrasodium salt (NADPH), l-Glutathione reduced, Glutathione reductase, Xanthine, Xanthine oxidase, Nitrotetrazolium blue chloride (NBT), Linoleic acid, Antioxidant assay kit, ABTS and dimethyl sulfoxide (DMSO) were obtained from Sigma-Aldrich, Co. (St. Louis, MO, USA). 2-Thiobarbituric (TBA) acid and 2-deoxy-d-ribose were obtained from Fluka (Darmstadt, Germany).

### 2.2. GC/MS Analyses

GC/FID and GC/MS analyses were carried out simultaneously on a Finnigan ThermoQuest TraceGC with a dual split/splitless injector, an FID detector and a Finnigan Automass quadrupole mass spectrometer. One inlet was connected to a 50 m × 0.25 mm × 1.0 μm SE-54 fused silica column (CS Chromatographie Service, Langerwehe, Germany), the other injector was coupled to a 60 m × 0.25 mm × 0.25 μm Carbowax 20 M column (J & W Scientific, Santa Clara, CA, USA). The two columns were connected at the outlet with a quartz Y connector and the combined effluents of the columns were split simultaneously to the FID and MS detectors with a short (*ca.* 50 cm) 0.1 mm ID fused silica restrictor column as a GC/MS interface. The carrier gas was helium 5.0 with a constant flow rate of 1.5 mL/min, injector temperature was 230 °C, FID detector temperature 250 °C, GC/MS interface heating 250 °C, ion source at 150 °C, EI mode at 70 eV, scan range 40–300 amu. The following temperature program was used: 46 °C for 1 min; 46 °C–100 °C at a rate of 5 °C/min; 100 °C–230 °C at 2 °C/min; 230 °C for 13.2 min. Compounds were identified using Finnigan XCalibur 1.2 software [21] with MS correlations with NIST [22], Adams Essential Oils [23], MassFinder [24]) and our own library. Retention indices of reference compounds and from literature data [25], ESO 2000 upd. 2006 (Leffingwell & Associates, GA, USA, 2006) were used to confirm peak data. Quantification of compounds was performed via peak area calculations of the FID chromatogram.

### 2.3. Antioxidant Activity *in Vitro*

The DPPH scavenging effect was determined according to Mensor *et al.* [26] 1 mL 0.3 mM ethanolic DPPH solution was added to 2.5 mL of the ethanolic juniper berry oil dilutions with different concentrations. The samples were kept at room temperature in the dark, and after 30 min the optical density of the samples, the blank or BHT as positive control was measured at 518 nm in comparison with ethanol.

The scavenging effect on radical cation ABTS^•+^ was determined using the Antioxidant Assay Kit (Sigma, CS0790, Saint Louis, MO, USA). Trolox, a water-soluble Vitamin E analog, serves as a standard or control antioxidant. BHT was used as positive control. One mL of the reaction mixture contained 10 µL ethanolic dilution of the juniper berry oil (1.5, 3.125, 6.25, 12.5, 25.0, 50.0 and 100 µg/mL), Twenty µL solution of myoglobin, 150 µL ABTS reagent (10 mL ABTS and 25 µL 3% H_2_O_2_). The samples were kept at room temperature in the dark, and after 10 min the optical density was measured at 405 nm.

Detection of ОН^•^ by deoxyribose assay was performed as described by Halliwell *et al.* [27] with minor changes. All solutions were freshly prepared. One mL of reaction mixture contained 28 mM 2-deoxy-d-ribose (dissolved in 50 mM phosphate buffer, pH 7.4), 500 µL of juniper berry oil dilution of various concentrations, 200 µM FeCl_3_ and 1.04 mM EDTA (1:1 v/v), 10 mM H_2_O_2_ and 1.0 mM ascorbic acid. After an incubation period of 1 h at 37 °C, the extent of deoxyribose degradation was measured by the TBA reaction. One mL TBA reagent (1% TBA in 50 mM NaOH) and 1.0 mL of trichloroacetic acid (TCA) were added to the reaction mixture and the tubes were heated at 100 °C for 20 min. After cooling, the absorbance was read at 532 nm against a blank (containing only buffer and deoxyribose). Quercetin was used as a positive control.

Superoxide anions were generated in an enzymatic system (xanthine-xanthine oxidase) and assayed by the reduction of NBT. The former comprised a solution of 100 µM xanthine, 60 µM NBT in 0.1 M phosphate buffer (pH 7.4) and 0.07 U/mL xanthine oxidase in a total volume of 1 mL. Before the enzyme was added, 0.025 mL of ethanolic dilution of juniper berry oil (100, 140,160, 180, 200, 240 and 280 µg/mL) were added to the samples. This mixture was incubated at 25 °C for 10 min, and the optical density was read at 560 nm against a blank without the enzyme [28]. In order to check the inhibitory effect of juniper berry oil on xanthine oxidase activity, the enzyme was assayed by measuring the formation of uric acid from xanthine [28].

The percentage inhibition was calculated using the following Formula 1: 
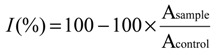
(1)

The IC_50_ represented the concentration of the compounds that caused 50% inhibition of radical formation.

For evaluation of antioxidant activity in the linoleic acid model system, linoleic acid emulsions were prepared by mixing 0.285 g linoleic acid, 0.289 g Tween 20 as emulsifier and 50 mL phosphate buffer (50 mM, pH 7.2). The mixture was homogenized for 5 min according to Yen *et al.* [29]. The antioxidant was added at the final concentrations of 0.02% (wt/v) of oil, BHT 0.01% was used as control. The mixture was incubated at 37 °C for 19 days. The course of oxidation was monitored by measuring the conjugated diene formation and TBA reactive substances (TBARS). The antioxidant activity at the end of the assay time was expressed as reduction percentage of peroxidation for each indicator. The control containing no antioxidant was 0%. A higher percentage indicates a higher antioxidant activity. For determination of conjugated diene formation, aliquots of 20 µL were taken at different intervals during incubation. After incubation, 2 mL of 60% methanol were added, and the absorbance of the mixture was determined at 233 nm [30]. A modified TBARS method was used to measure the antioxidant activity of oil in terms of inhibition on lipid peroxidation. 0.1 mL of sample was taken from the emulsion every day, and the following were sequentially added: the TBA-TCA solution (20 mM TBA in 15% TCA). The mixture was heated at 100 °C for 15 min and cooled at room temperature. After adding 2 mL of chloroform, the mixture was mixed and centrifuged at 2000 rpm for 15 min. The chloroform layer was separated and the absorbance of the supernatant was measured at 532 nm against a blank containing TBA-TCA solution [31].

For hydrogen peroxide scavenging activity, juniper berry oil dilutions (50, 100, 200, 400 and 800 µg/mL, dissolved in 0.0125, 0.025, 0.05, 0.1 and 0.2 mL DMSO, respectively) were added to 1 mL 20 mM Н_2_О_2_ in phosphate buffer (0.1 M, pH 7.3). The initial and the final absorbance of the samples after an incubation period of 1 h at 25 °С were measured at 240 nm. Controls containing 20 mM Н_2_О_2_ and the same DMSO volumes were prepared in a similar way. Hydrogen peroxide concentration was determined according to the Formula 2:

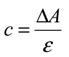
(2)

Δ*А* = the difference in absorbance at the end and at the beginning of reaction; ε = molar absorptivity of Н_2_О_2_ = 43.6 M^−1^ cm^−1^.

### 2.4. Antioxidant Activity *in Vivo*

For the *in vivo* analyses, *S. cerevisiae* from the collection of the Department of Biotechnology at the University of Food Technologies, Plovdiv, were used. The strain was cultivated aerobically in a liquid medium (1% yeast extract, 1% Bacto-peptone, 2% glucose) for 48 h at 30 °C. The cells were centrifuged (3000 min^−1^), washed with phosphate buffer (50 mM, pH 7.0) and centrifuged again. Then, they were resuspended and diluted in phosphate buffer, pH 7.0 resulting in a final optical density at 600 nm of 0.256.

For the assays for antioxidant enzymes in yeast cells subjected to oxidative stress with hydrogen peroxide, 1 mM H_2_O_2_ (final concentration) was added to 1 mL of yeast suspension, which was then incubated for 16 h in the dark with periodic shaking. The yeast cells were centrifuged at 4000 min^−1^, washed twice with phosphate buffer (50 mM, pH 7.0), centrifuged and resuspended in phosphate buffer to 1 mL.

The reaction mixture for determination of the activities of antioxidant enzymes in the presence of various concentrations of the essential oil contained 1 mL suspension (1.39 × 10^7^ CFU/mL), 1 mM H_2_O_2_ (final concentration) and juniper berry oil in different concentrations (0.4, 0.8, 1.6, 3.2 and 4.0 mg/mL added in 0.01, 0.02, 0.04, 0.08 and 0.1 mL DMSO, respectively). Yeast suspension controls were also prepared using the same DMSO volumes. The samples were incubated for 16 h in the dark with periodic shaking. Then, the yeast cells were centrifuged at 4000 min^−1^ for oil and DMSO removal, washed twice with phosphate buffer (50 mM, pH 7.0), centrifuged and resuspended in phosphate buffer to 1 mL. This whole cell suspension was used for the evaluation of the enzymes superoxide SOD, CAT and GPx. The resultant enzyme activities were compared to those of *S. cerevisiae* cells not treated with oil. For evaluation of the protein content in yeast cells, the suspension was subjected to heat treatment for 20 min at 60 °С. The resultant cell lysate was centrifuged at 4000 min^−1^ and the protein in the supernatant was determined according to the Lowry method [32].

SOD (EC 1.15.1.1) activity was assayed by the NBT test [33]. NBT was reduced to blue formazan by ^•^O_2_^−^, which has a strong absorbance at 560 nm. The presence of SOD inhibited the reaction. The assay mixture consisted of sodium carbonate buffer (pH 10.2) containing xanthine, NBT, EDTA and 25 µL of yeast suspension (1.39 × 10^7^ CFU/mL). The reaction was initiated by the addition of 50 µL xanthine oxidase (0.1 mg/mL) and the mixture was incubated for 30 min at room temperature. The reaction was stopped by adding 6 mM copper(II) chloride and the mixture was centrifuged at 1500 rpm for 10 min. The absorbance of blue formazan in the supernatants was measured at 560 nm. One unit of SOD was defined as the enzyme amount causing 50% inhibition in the NBT reduction. Activity was expressed as units per mg protein.

CAT (EC 1.11.1.6) activity was measured according to Carillo *et al.* [34]. The decomposition of H_2_O_2_ 3% (v/v) was monitored by a decrease in absorbance at 240 nm. The assay mixture contained 25 µL of yeast suspension (1.39 × 10^7^ CFU/mL) in 50 mM phosphate buffer (pH 7.0) at a final volume of 1.0 mL. The samples were incubated for 2 min at 37 °C and the absorbance of the samples was monitored for 3 min. One unit of CAT was defined as the enzyme amount causing decomposition of 1 µmol H_2_O_2_ in 1 min.

GPх (EC 1.11.1.9) activity was assayed by the method of Paglia *et al*. [35]. The reaction mixture contained 0.1 M phosphate buffer (pH 7.0), EDTA, glutathione, NaN_3_, 1 unit of glutathione reductase, 1.5 mM NADPH and 25 µL of yeast suspension (1.39 × 10^7^ CFU/mL). After incubation for 10 min at 37 °C, H_2_O_2_ was added to each sample at a final concentration of 20 mM. The GPx activity was measured as the rate of NADPH oxidation at 340 nm. One unit of GPx was defined as the enzyme amount causing oxidation of 1 µmol NADPH in 1 min.

CAT and GPx enzyme activities (EA) were expressed as units per mg protein (U/mg) and calculated according to the Formula 3:

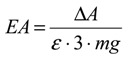
(3)

Δ*А* = the difference in absorbance at the end and at the beginning of reaction; ε = 0.0436 μmol^−1^ cm^−1^ of Н_2_О_2_ for CAT; ε = 6.3 μmol^−1^ cm^−1^ of NADPH for GPx; 3 = reaction time (min).

### 2.5. Statistical Analysis

The experimental data analysis included approximation via fourth order polynomial dependences. For all cases, the plural correlation coefficient *R*^2^ was determined. The concentration level corresponding to 50% of inhibition was calculated according to this approximated dependence for which *R*^2^ was the maximum. The mathematical analysis of the data was carried out with MATHLAB [36]. All values of the enzyme activities are presented as mean ± SD (*n* = 3). The statistical differences between the activities of the treated and untreated yeast, and between oil-treated yeast and yeast-treated with DMSO were analyzed by Student’s *t*-test. Differences showing *p* ≤ 0.05 were considered statistically significant.

## 3. Results and Discussion

### 3.1. Chemical Composition of Juniper Berry Oil

Using GC/FID and GC/MS, 70 compounds were identified in the essential *Juniperus communis* L. oil (altogether, about 96% of the volatiles). As main components, the monoterpene hydrocarbons α-pinene (51.4%), myrcene (8.3%), sabinene (5.8%), limonene (5.1%) and β-pinene (5.0%) were found (Table 1). Concluding, the essential oil mainly comprises mono- and sesquiterpene hydrocarbons (80.4% and 9.6%, respectively); oxygenated derivatives are only minor constituents of this essential oil.

**Table 1:** Chemical composition of juniper berry oil.

Substance	Retention Index	% Area
toluene	771	trace
hexanal	800	trace
α-thujene	933	0.9
α-pinene	943	51.4
α-fenchene	957	0.2
camphene	959	0.8
thuja-2,4(10)-diene	964	0.2
sabinene	981	5.8
β-pinene	988	5.0
myrcene	992	8.3
δ-3-carene	1019	0.2
α-terpinene	1024	0.1
p-cymene	1032	0.9
limonene	1037	5.1
β-phellandrene	1038	0.5
(*E*)-β-ocimene	1050	0.1
γ-terpinene	1065	0.2
*cis*-sabinene hydrate	1075	0.1
terpinolene	1096	0.4
linalool	1101	0.1
perillene	1104	0.1
trans-sabinene hydrate	1107	0.1
α-pinene oxide	1111	0.1
*trans*-p-menth-2-en-1-ol	1131	tr
campholen aldehyde	1136	0.1
*trans*-pinocarveol	1154	0.3
*cis*-verbenol	1156	0.5
borneol	1180	trace
terpinen-4-ol	1189	0.9
*p*-cymen-8-ol	1193	0.2
α-terpineol	1201	0.2
myrtenol	1208	0.1
myrtenal	1210	0.2
verbenone	1223	0.2
*trans*-carveol	1228	0.2
carvone	1256	0.1
methyl citronellate	1260	0.1
undecan-2-one	1294	0.1
bornyl acetate	1298	0.3
methyl geranate	1326	trace
citronellyl acetate	1352	0.1
terpinyl acetate	1359	0.1
α-cubebene	1366	0.6
geranyl acetate	1382	trace
α-copaene	1397	0.5
β-elemene	1409	0.7
α-gurjunene	1426	0.2
longifolene	1438	0.1
(*E*)-β-caryopyhllene	1447	2.0
γ-elemene	1451	0.1
(*E*)-β-farnesene	1461	0.3
α-humulene	1481	1.3
γ-muurolene	1497	0.4
α-amorphene	1501	0.1
germacrene D	1507	1.1
β-selinene	1515	0.2
α-muurolene	1520	0.4
α-selinene	1521	0.2
γ-cadinene	1538	0.5
δ-cadinene	1543	0.8
α-cadinene	1560	0.1
spathulenol	1607	0.3
caryophyllene oxide	1616	0.9
humulene epoxide II	1643	0.5
τ-muurol + τ-cadinol	1668	0.2
α-cadinol	1681	0.1
*m*-camphorene	1964	0.2
*p*-camphorene	2002	0.1
sum		96.0

For comparison, Estonian juniper berry oil is also dominated by α-pinene (47.9%) [37], whereas the essential oil from *Juniperus communis* ssp. *hemisphaerica* is dominated by sabinene (25.1%) and α-pinene (13.6%) [6]. Regardless of the domination of monoterpene compounds in the oils, there are differences in their quantitative composition due to a number of factors: geographical location, degree of ripeness and age, production method, *etc*. These differences underlie the individual biological properties of juniper berry essential oils.

### 3.2. Antioxidant Activity of Juniper Berry Oil *in Vitro*

DPPH assay was one of the *in vitro* tests used in this study to determine the ability of juniper berry oil components to act as hydrogen atom donors. It is usually regarded as a reaction of hydrogen atom transfer, but on the basis of the kinetic data, an electron transfer mechanism can also be considered for this assay [38,39]. Juniper berry essential oil was a weak DPPH radical reducer with IC_50_ value of 34.80 mg/mL (*R*^2^ = 0.9896), compared to BHT (IC_50_ = 4.414 μg/mL, *R*^2^ = 0.999). Limonene in 10–50 μg/mL concentrations causes DPPH inhibition from 16% to 25% [12]. Emami *et al.* [6] established that γ-terpinene (17.74%) showed antiradical activity in relation to DPPH radicals, while β-pinene had extremely low activity (0.96%), and of α-pinene had no activity.

The antioxidant assay principle ABTS radical cation scavenging activity is the formation of a ferryl myoglobin radical from metmyoglobin and H_2_O_2_, which oxidizes the ABTS to produce a radical cation, ABTS^•+^. Antioxidants suppress the production of the radical cation in a concentration dependent manner and the color intensity decreases proportionally. Juniper berry essential oil showed a significant inhibitory effect of ABTS radicals (IC_50_ = 10.96 µg/mL, *R*^2^ = 0.9048), but again, BHT was considerably stronger (IC_50_ = 0.0175 µg/mL, *R*^2^ = 0.9996).

The results of the deoxyribose degradation inhibition also showed other action mechanisms of the antioxidants in juniper berry essential oil. During incubation of Fe^3+^-EDTA with H_2_O_2_ and ascorbic acid at pH 7.4, ОН^•^ were formed, which was indicated by 2-deoxy-d-ribose degrading to fragments yielding a pink color when heated with TBA at low pH [27]. The juniper berry oil added to the reaction mixture removed the ОН^•^ from the sugar and protected it against degradation. The effect of the inhibition of ОН^•^ by juniper berry oil was expressed by IC_50_ = 0.0066 µg/mL (*R*^2^ = 0.9199), which was 931.18 times higher than that of quercetin (IC_50_ = 6.15 µg/mL, *R*^2^ = 0.996). In the absence of EDTA in the reaction mixture, some of the Fe^3+^ ions were able to form a complex with deoxyribose and participate in the formation of OH^•^. Only the molecules which can chelate Fe(III) and form a more stable complex with Fe(III) than ETDA and inactivate them can inhibit deoxyribose degradation. This action mechanism of juniper berry oil was proved in our studies. Juniper berry oil showed significant chelating capacity with IC_50_ of 1.083 µg/mL (*R*^2^ = 0.9512), fully comparable to ОН^•^ scavengers. The chelating capacity of the oil was 5.72 times stronger than that of quercetin with IC_50_ of 6.2 µg/mL (*R*^2^ = 0.999). Regarding the deoxyribose degradation assay, Emami *et al*. [6] established the strongest effect for pure compounds β-pinene and limonene. The *Juniperus oblonga* berry oil demonstrated the strongest anti-radical effect which, as the authors believe, may be attributed to the large amounts of β-pinene (20.8%) in oil. Establishing the chelation of Fe(III) by juniper berry essential oil is important for our research using the *S. cerevisiae* model organism. The main source of OH^•^ radical production was the Fenton reaction which occurred between Fe^2+^ and H_2_O_2_. Srinivasan *et al.* [40] showed that in yeast (wild-type and sod mutants), unlike in *E. coli* and mammals cells, most, if not all, EPR-detectable iron (free iron) was present in the Fe(III) state. On the other hand, excess superoxide could generate iron reduction by the Haber-Weiss reaction and, in turn, the ferrous ion could take part in the Fenton reaction.

Superoxide is biologically important since it can be decomposed to form stronger ROS such as singlet oxygen and ОН˙. Superoxide anions indirectly initiate lipid oxidation as a result of superoxide and hydrogen peroxide serving as precursors of singlet oxygen and ОН^•^ [28]. Results from the *in vitro* experiments on the scavenging activity on ^•^O_2_^−^ and the inhibitory effect on xanthine oxidase activity proved that the juniper berry essential oil, introduced into the reaction mixture, scavenged ^•^O_2_^−^ radicals with an IC_50_ of 0.822 µg/mL (*R*^2^ = 0.9805) in a similar manner as SOD but using mechanism different from the enzyme. As an antioxidant, this essential oil possesses a second mechanism of action—to inhibit the activity of xanthine oxidase, which led to decreased production of ^•^O_2_^−^ radicals (IC_50_ 176.38 µg/mL, *R*^2^ = 0.9938).

**Figure 1:**
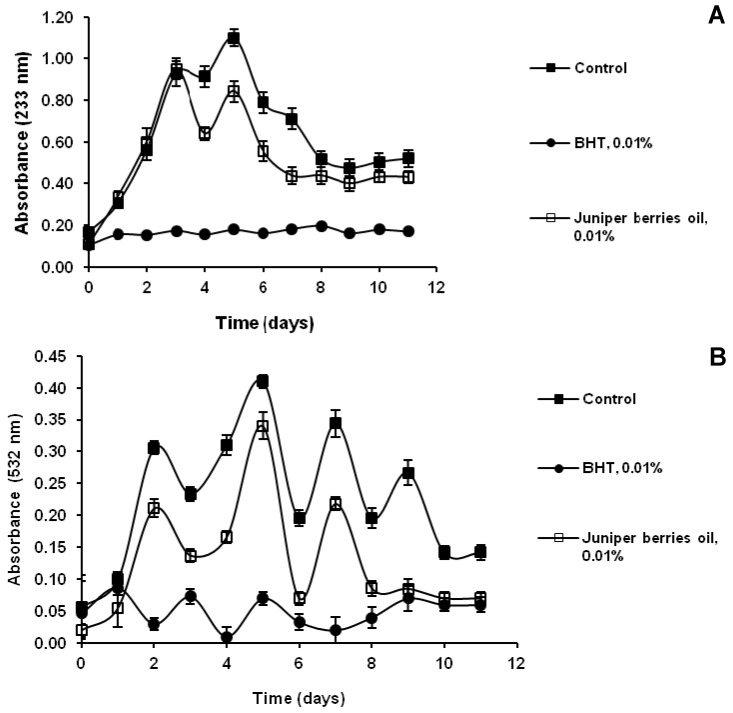
Effect of juniper berry oil on (**A**) conjugated dienes and (**B**) TBARS in a linoleic acid/water emulsion system.

An important mechanism of antioxidant activity is the inhibition of linoleic acid oxidation. Polyunsaturated fatty acids such as linoleic acid are easily oxidized by atmospheric oxygen. This auto-oxidation leads to chain reactions with formation of conjugated double bonds and by-products such as aldehydes, ketones and alcohols. The unoxidized linoleic acid molecules have two unconjugated double bonds and no absorbance at 233 nm. During the oxidation of lipid molecules, conjugated double bonds are formed, whereby lipid peroxides and hydroperoxides are produced, their absorbance at 233 nm increasing in relation to their concentration. Linoleic acid peroxidation caused by the formation of conjugated double bonds showed two absorbance maximums: on the third and the fifth day of incubation (control in this study) (Figure 1A). In the samples containing juniper berry oil in 0.01% (wt/v) concentration, 23.63% inhibition was observed at the second peak of peroxide production, followed by a period of attenuation in the formation of lipid peroxides and hydroperoxides. Inhibition at the first peak is not observed.

The use of TBA reagent showed the presence of malonaldehyde—a secondary product of the linoleic acid peroxidation—which yielded a pink colored product with absorption maximum at 532 nm. The control in this study showed four peaks in the formation of lipid peroxidation by-products: on the second, fifth, seventh and ninth day of the study (Figure 1B). The addition of juniper berry oil to the reaction emulsion reduced significantly the formation of lipid peroxidation by-products. On the second day of the process, 31.93% inhibition of lipid peroxidation was achieved; on the fifth day it was 35.12%, on the seventh and ninth day, 35.88 and 68.51%, respectively. Juniper berry oil inhibited to a larger extent the second of the two lipid peroxidation mechanisms, *i.e.*, conjugated double bond formation and production of by-products of linoleic acid. It was less efficient than BHT in both processes of lipid peroxidation inhibition. Ruberto *et al.* [10] proved that α- and γ-terpinene and terpinolene had the highest antioxidant activity in both lipid peroxidation stages, the activity of α- and γ-terpinene being comparable to that of α-tocopherol. α-Pinene, sabinene and limonene exhibited weak activity only at the stage of by-product formation. The degree of lipid peroxidation inhibition by the juniper berry essential oil studied was determined by its composition. The oil was dominated by α-pinene (51.4%) and myrcene (8.3%), with considerably lower concentration of terpinolene (0.4%), α-terpinene (0.1%) and γ-terpinene (0.2%) as the carriers of higher antioxidant activity.

**Figure 2:**
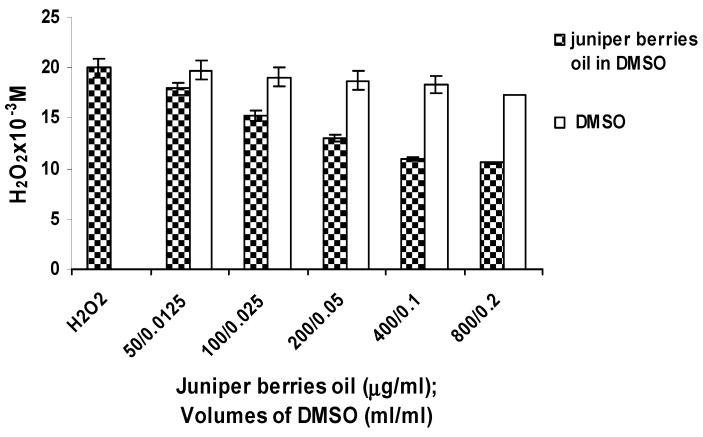
Hydrogen peroxide scavenging activity of juniper berry oil.

Hydrogen peroxide scavenging activity of juniper berry oil was observed at concentrations from 50 to 800 µg/mL within the whole range studied (Figure 2). The initial concentration of 20 µМ Н_2_О_2_ decreased to 10.54 µМ in the presence of 800 μg/mL essential oil after 30 min of action. Thus, the oil imitated CAT action, the substrate for its action being Н_2_О_2_. Under enzyme action, however, the enzyme remained unchanged whereas the juniper oil action in relation to hydrogen peroxide was probably due to the oxidation of some of the oil components. These results were consistent with [8,41], which proved that cyclic monoterpene hydrocarbons α- and γ-terpinene (contained in juniper berry essential oil) were oxidized to the aromatic hydrocarbon p-cymene. The DMSO solvent used in increasing volumes had a weak effect on Н_2_О_2_, the largest input volume of 0.2 mL reducing its concentration by 15.80%. Gülçin *et al.* [42] also reported scavenging activity of clove oil on Н_2_О_2_. The hydrogen peroxide scavenging property of essential oil is of great biological significance. Hydrogen peroxide is not a free radical but can generate the exceptionally strong ОН^•^. Furthermore, Н_2_О_2_ easily diffuses through mitochondrial membranes and can oxidize various compounds [43,44].

The nine tests used for *in vitro* evaluation of juniper berry essential oil demonstrated its different action mechanisms. Its hydrogen atom (electron) donating capacity was proven by the DPPH assays and lipid peroxidation inhibition in both its stages. The investigated oil also had an electron yielding capacity—a mechanism underlying in OH^•^, ABTS^•+^, ^•^O_2_^−^ scavenging and OH^•^ formation (chelating capacity). The antioxidant activity which was due to electron transfer made juniper berry essential oil a strong antioxidant. The antioxidant activity of the oil in descending order is: OH^•^ > xanthine oxidase inhibitory effects > chelating capacity > ABTS^•+^ > ^•^O_2_^−^. The antioxidant activity of the oil attributable to hydrogen atom transfer (DPPH assay) and lipid peroxidation inhibition were lower compared to BHT as standard. A number of researchers believe that the data on the antioxidant activity of essential oils or their components obtained according to different methods are practically incomparable. This is due both to the difference in the protocols used and to the different composition of the essential oils studied.

### 3.3. Action of Juniper Berry Essential Oil on the Antioxidant Protection of *Saccharomyces cerevisiae* Model Organism

**Figure 3:**
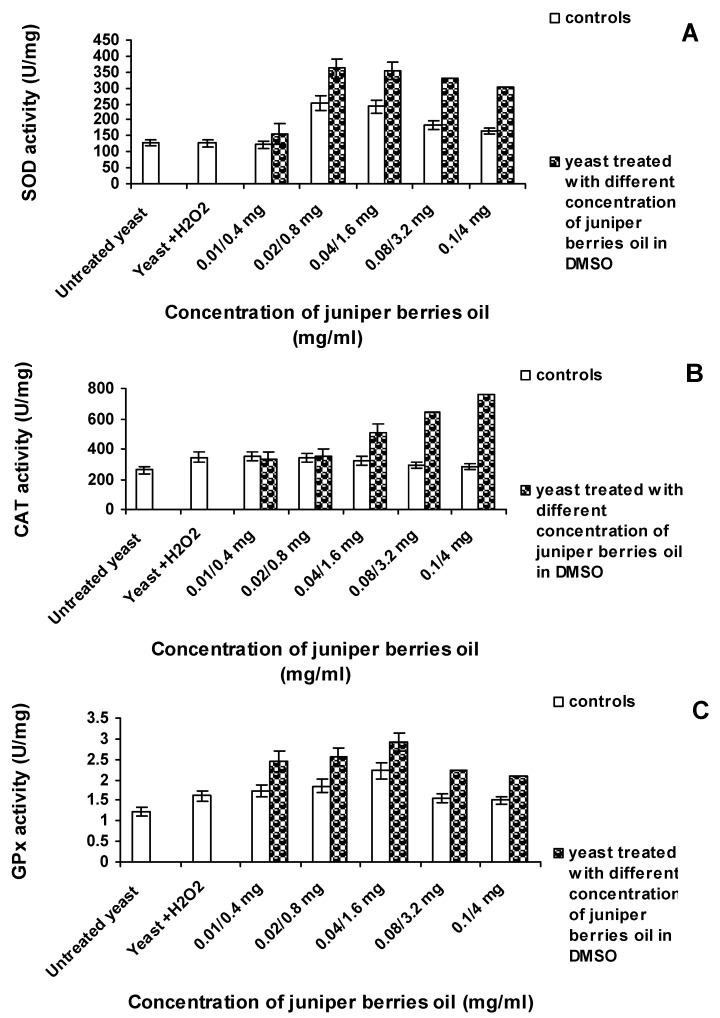
Effect of juniper berry oil on (**A**) superoxide dismutase (SOD), (**B**) catalase (CAT) and (**C**) glutation peroxidase (GPx) activity in *S. cerevisiae* cells.

In this aspect, the essential oil effect on whole cells of wild *S. cerevisiae* strain was studied. It was evaluated *in vivo* on the antioxidant enzymes SOD, CAT and GPx (Figure 3). Molecular oxygen is assimilated as a substrate by the living cells and participates in different reactions catalyzed by the enzymes oxygenase, oxidase and hydroxylase. All these enzymes work interrelatedly, and the study of exogenous antioxidants upon certain enzymes in whole cells would provide an evaluation being as close as possible to the metabolic processes occurring in the cells. Oxygen assimilation is at the expense of partially reduced oxygen species, including the production of free radicals: ^•^O_2_^−^, Н_2_О_2_ and ОН^•^. In the current experiment, enzyme levels of *S. cerevisiae* cell suspension were established before and after 16 h treatment with different essential oil concentrations. During that period, the cells were also subjected to starvation.

For SOD evaluation, in the whole yeast cells, the xanthine-xanthine oxygenase system generating ^•^O_2_^−^ was used. The addition of whole cells only in the presence of xanthine caused xanthine oxidation under the effect of their own xanthine oxidase. Therefore, for the evaluation of SOD as control, the xanthine-xanthine-oxidase system and heat-inactivated cells (for elimination of the action of their own xanthine oxygenase) were used. The evaluation of CAT using whole cells was facilitated by the fact that the substrate for the action of this enzyme, *i.e.*, Н_2_О_2_ was non-ionized and easily diffused through the hydrophobic membranes of the mitochondrial biological membranes [43,44]. SOD participates in the dismutation of ^•^O_2_^−^ in hydrogen peroxide and molecular oxygen. CAT only decomposed Н_2_О_2_, and yeast GPx acted both on H_2_O_2_ and organic hydroperoxides. The influence of oxidative stress (1 mM H_2_O_2_) on yeasts cells (Figure 3) could be demonstrated by increased levels of CAT (342.2 U/mg) and GPx (1.61 U/mg) activities, which were 1.58 and 1.32 times, respectively, higher compared to untreated yeast. SOD activity remained the same (128.20 U/mg).

The increase in the CAT activity in yeast cells treated with H_2_O_2_ can be explained by the inducible transcription of the CTT1 gene encoding CAT in *S. cerevisiae* cytoplasm [45,46]. It has also been proved that under oxidative stress, GPx genes of *S. cerevisiae* also encode phospholipid hydroperoxide GPx and that these enzymes protect yeast against phospholipid hydroperoxides [20,47]. In *S. cerevisiae*, the oxidative stress of Н_2_О_2_ did not influence the expression of the genes, encoding Cu/Zn SOD and Mn SOD [46]. Yeast cells treated with juniper berry essential oil exhibited direct dependence of the three enzymatic activities on the essential oil concentration (Figure 3).

In further experiments, it was examined if the level and concentration of the essential oil introduced into yeast suspension subjected to an oxidative stress, influenced *in vivo* the SOD activity in the yeast cells (Figure 3A). After 16-h oxidative effect and increasing concentrations of the essential oil from 0.4 to 0.8 mg/mL, SOD activity increased respectively from 156.34 to 361.29 U/mg. Introduction of higher concentrations of the essential oil (1.6, 3.2 and 4.0 mg/mL) caused a decrease in SOD activity to 304.84 U/mg (4 mg/mL). However, this enzyme activity is still 2.38 times higher compared to the control (128.23 U/mg).

In contrast to SOD, the results for the CAT showed continual increase of the enzyme activity with increasing concentrations of the introduced essential oil (Figure 3B). The influence of the essential oil is most significant on the CAT activity in the cells at a concentration of 4.0 mg/mL (756.22 U/mg). The resulting activity is 2.91 times higher than the activity of the cells not subjected to oxidative stress (260.03 U/mg) and 2.21 times higher than the activity of the cell subjected to oxidative stresses (342.42 U/mg).

Results for the GPx activity in the yeast cells (Figure 3C) showed that the highest activity of 2.92 U/mg can be achieved at a concentration of 1.6 mg/mL of the juniper berry essential oil. Below and above this concentration, GPx activity was lower. GPx activity reached at a concentration of 1.6 mg/mL was 2.39 times higher than the activity of the untreated yeast cells (1.22 U/mg), and 1.81 times higher than that in the experiment (yeast + H_2_O_2_) (1.61 U/mg). The decrease in the GPx activity at concentrations of the essential oil above 1.6 mg/mL could be explained by the fact that, at those concentrations, CAT activity increases because of the same substrate activity. It was obvious that DMSO influenced the levels of the enzymes examined. Within the studied range, SOD and GPx are active only in the presence of the solvent DMSO, and these activities change in the presence of the essential oil. However, as absolute values, these activities are lower than those obtained in the presence of the essential oil. DMSO had the least impact on the CAT activities. Activities of SOD (Figure 3A), CAT (Figure 3B) and GPx (Figure 3C) of yeast treated with juniper berry were significantly (*p* < 0.05) different compared to controls (untreated yeast) and yeast treated with DMSO.

Under starvation conditions and treatment with juniper essential oil, yeast cells exhibited higher antioxidant capacity than the antioxidant protection of cells subjected to oxidative stress by 1 mM Н_2_О_2_. The maximum SOD, CAT and GPx activities were, respectively, 2.82, 2.21 and 1.81 times higher than the activities of the activities of those enzymes treated with H_2_O_2_. SOD is the first protection line against oxidative stress in living organisms [48]. The scavenging of ^•^O_2_^−^ which is a precursor of highly reactive ROS such as ОН^•^, is particularly important for organism adaptation under oxidative stress. Considering the fact that the expression of the genes encoding Cu/Zn SOD and Mn SOD in *S. cerevisiae* is not inducible by oxidative stress [46], as our *in vitro* study proved that juniper berry essential oil introduced into reaction mixture is able to scavenge ^•^O_2_^−^ radicals and to inhibit xanthine oxidase, we can assume that the increasing SOD activity resulted from the action of juniper berry essential oil. We proved *in vitro* that juniper oil could degrade hydrogen peroxide similarly to CAT action (Figure 2). Monoterpene compounds are known to be able to penetrate cells [7] and therefore neutralize endogenous Н_2_О_2_. The significant increase in the CAT and GPx activity in yeast cells in the presence of juniper essential oil and under starvation stress conditions may be due to inducible gene transcription [20,45,46,47] and probably to a larger extent to the CAT-like action of the oil components.

The enhanced antioxidant protection was indirect evidence of the change in the endogenous levels of H_2_O_2_ and other organic peroxides in this microorganism. Higher enzyme activities in the yeast cells meant better ability of the cells to degrade hydrogen peroxide, organic hydroperoxides and phospholipid hydroperoxides. Thus, it would follow that the level of these peroxides within the cells would be lower at higher enzyme activity. Roberto *et al.* [12] proved that a lower Н_2_О_2_ endogenous level corresponded to a higher CAT and GPx activity in lymphocyte cells treated with the monoterpene compound limonene. If the increasing activity of these enzymes enables cells to neutralize the ROS, their decreasing activity should be considered as a decreasing ability of cells to neutralize them. The treatment with high concentrations of essential oils (4 mg/mL) probably induced the damage to *S. cerevisiae*. Parveen *et al*. [49] reported damages induced to *S. cerevisiae*. The authors found that the *S. cerevisiae* participating in ergosterol biosynthesis and assimilation, lipid metabolism, cell wall structure and function, and cellular transport were affected by α-terpinene treatment.

## 4. Conclusions

*In vitro* antioxidant research of juniper essential oil proved the existence of several mechanisms which enabled radical scavenging, Н_2_О_2_, the prevention of radical formation (chelating capacity, inhibitory effect on xanthine oxidase) and protection against lipid peroxidation. *In vivo* studies confirmed these effects of the oil which created the possibility of blocking the oxidation processes in yeast cells and enhanced their adaptivity to ROS. The biological effects of juniper berry essential oil *in vivo* were directly dependent on the concentrations applied. It is well known that ROS contribute to organism aging and the etiopathogenesis of various diseases. The proved ability of juniper berry essential oil to enhance adaptivity to ROS *in vivo* adds new details to the essential oil properties. These properties determine its potential for food additive production, an efficient way to improve people’s health and quality of life. Furthermore, it expands its areas of application to perfumery, cosmetics, pharmacy and medicine.
